# Deep Sequencing Reveals Novel Mutations in *Androgen Receptor*-Related Genes in Prostate Cancer

**DOI:** 10.3390/ijms26188758

**Published:** 2025-09-09

**Authors:** Abraham Pedroza-Torres, Noemí Baranda-Avila, Jorge L. Ramírez, Maricruz González, Pamela A. González, Blanca L. Torres, Miguel A. Jiménez-Ríos, Alfonso Méndez-Tenorio, Rosa María Álvarez-Gómez, Greco Hernández

**Affiliations:** 1 Cátedra SECIHTI-Clínica de Cáncer Hereditario, National Institute of Cancer (Instituto Nacional de Cancerología, INCan), Mexico City 14080, Mexico; apedrozato@conacyt.mx; 2 Laboratory of mRNA and Cancer, Unit of Biomedical Research on Cancer, National Institute of Cancer (Instituto Nacional de Cancerología, INCan), Mexico City 14080, Mexico; mimi19481@yahoo.com.mx (N.B.-A.);; 3 Department of Oncologic Urology, National Institute of Cancer (INCan), Mexico City 14080, Mexico; 4 Laboratory of Biotechnology and Genomic Bioinformatics, National School of Sciences, National Polytechnic Institute (Instituto Politécnico Nacional), Mexico City 07840, Mexico; 5 Clínica de Cáncer Hereditario, National Institute of Cancer (Instituto Nacional de Cancerología, INCan), Mexico City 14080, Mexico; 6 Tecnologico de Monterrey, Escuela de Medicina y Ciencias de la Salud, Mexico City 14380, Mexico

**Keywords:** prostate cancer, *Androgen Receptor*, *FOXA1*, *SPOP*, *TP53*

## Abstract

Prostate cancer (PCa) is the second most frequent tumor and the fifth leading cause of cancer-related death in men worldwide. PCa shows the largest clinical disparities across Asian, Caucasian, and African descendants among all cancer types, proving that the ethnic genetic background plays a significant role in PCa. *Androgen Receptor* (*AR*) gene malfunctioning represents the most prevalent cause of PCa. *AR* also displays a broad spectrum of genetic variability across ethnic backgrounds differently associated with cancer risk. We conducted a massive sequencing analysis of 15 genes highly relevant for PCa or the AR activation pathway in biopsies from 64 tumors and 36 benign prostate samples from Mexican patients. We identified 3414 genomic mutations and observed that *AR*, *SPOP*, *TP53*, *FOXA1*, and *MTOR* had the highest rate of pathogenic mutations in tumors, evidencing their relevance in PCa. *AR* showed 13 unique mutations, followed by *SPOP* (6), *TP53* (5), *FOXA1* (4), and *MTOR* (3). We discovered 19 novel mutations specific of Hispanic patients, a population only scarcely studied, thus adding critical information on the genetic diversity of the mutational landscape in genes key for PCa. We discuss the clinical relevance of these mutations and predict the structural consequences on the proteins. Mutations in *FOXA1* showed significant negative association with patient survival and might be used as novel PCa markers, at least for Hispanic men.

## 1. Introduction

Prostate cancer (PCa) is the second most frequently diagnosed tumor in men and the fifth cause of death by cancer worldwide. About 2.9 million new cases and 795,000 deaths from this type of tumor were reported in 2022, and it is expected that the PCa incidence will be duplicated between 2020 and 2040. Moreover, deaths will increase by about 85%, particularly in low-income countries [1,2,3,4]. PCa results from the uncontrolled proliferation and dispersion of cells from the prostate epithelium. Indeed, PCa is a heterogeneous illness that displays a broad spectrum of clinical evolution and phenotypic defects [5,6,7]. Currently, several factors limit the diagnosis, prognosis, and treatment of PCa in the early stages. On the one hand, the clinical management of prostate tumors has demonstrated extreme complexity due to the high heterogeneity of factors such as age, race/ethnicity, and family history, which modify the clinical outcome of PCa patients [7,8,9,10]. PCa has one of the largest clinical disparities among races of all cancer types. PCa comparative studies mostly among Caucasian, Asian, and African descendants have proven that the race/ethnic genetic background plays a significant role in the origin and progression of this disease [10,11,12]. On the other hand, the wide use of the blood level of prostate-specific antigen (PSA) as a diagnostic marker has enhanced patients’ early detection of PCa. However, its predictive value is currently under debate, as PSA is frequently elevated in patients with benign tumors, hyperplasia, and prostatitis, limiting its specificity for cancer [7,13], and because it possesses poor diagnosis and predictive values of PCa mortality in population screenings [14,15]. Thus, new research has favored the use of other methods for early detection of cancer such as the Gleason score of tumors, the Prostate Health Index (phi), and the urinary PCA3 level with slightly better results [7,16,17].

At the molecular level, the gene fusion *TMPRSS2-ERG* is one of the most conspicuous tumor signatures [18], and genome-wide association studies have identified alterations (i.e., point mutations, chromosome rearrangements, indels, and copy number variations) in more than 100 low-penetrance *loci* defining the molecular landscape of PCa [19]. However, so far, they have shown minimal power of predicting cancer risk. Misexpression of other *loci* with high penetrance have also been associated with PCa, including long non-coding (Inc)RNA RPS-997D16.2, *AR*, *BRCA2*, *HOXB13*, *TP53*, *RB1*, *PTEN*, *TMPRSS2-ERG* fusion, *ETV1*, *ETV4*, *FLI1*, *SPOP*, *FOXA1*, *IDH1*, *BRCA2*, *ATM*, *MYC*, and *BRAF.* Although they might have clinical utility, their possible value in predicting cancer risk and treatment options remain under study [19,20,21,22,23,24,25]. The differential occurrence of mutations among Asian, Caucasian, and African descendants has also been observed for some genes, including *SPOP* [26], the gene fusion *TMPRSS2-ERG* [27,28,29], p16 [30], the microRNA mir34 [31], and *FOXA1* [32].

Malfunctioning of the transcription factor *Androgen Receptor* (*AR*) represents the most prevalent cause of onset and progression of PCa, as the prostate function strongly depends on the action of both androgens and AR. AR is part of the family of steroid hormone receptors and regulates hundreds of genes responsible for the development of male characteristics [33,34,35,36]. Accordingly, 2–18% of prostate tumors show point mutations in the *AR* gene, and gene amplifications have been reported in 5–52%, preferably in castration-resistant PCa subtypes [12,21,22,37,38,39,40]. The *AR* gene also displays a broad spectrum of variability in both polymorphisms and mutations across different ethnical backgrounds that might be differently associated with cancer risk [41,42,43,44,45]. The relationship between *AR* mutational variability with mutations in other genes and their clinical outcome is also scarcely understood.

To better understand the role that *AR* gene mutagenesis plays in prostate carcinogenesis, we conducted a massive, next-generation sequencing (NGS) analysis of a panel of genes highly relevant for PCa in prostate tumor biopsies from a scarcely studied cohort, namely Hispanic patients. Our results showed that *AR*, *SPOP*, *TP53*, *FOXA1*, and *MTOR* carried a high rate of pathogenic mutations in patients with CaP, evidencing their prostate cancer relevance. Among them, we report 19 novel mutations, correlate them with the clinical profiles of patients, and discuss their possible impact in PCa. We also discovered that mutations in *FOXA1* were significantly associated with patient survival.

## 2. Results

### 2.1. Clinicopathologic Characteristics of Patients

We analyzed biopsies from 160 patients who attended the Department of Oncologic Urology of the National Institute of Cancer (Mexico City) between February 2016 and September 2018. Overall, 96 (60%) patients met the described criteria to be included in the NGS analysis. The patient inclusion criteria were as follows: Mexican men with all four Mexican grandparents; patients who have signed informed consent; men ≥ 18 years of age with the capacity to make their own decisions; histologically or cytologically confirmed diagnosis of prostate carcinoma without neuroendocrine differentiation or small cell features; any Gleason score; adequate organ function; any TNM stage; and ECOG score of 0 or 1. The exclusion criteria were as follows: patients with symptomatic local or regional disease requiring medical intervention; those who have received any treatment prior to biopsy collection; comorbidity with another type of cancer, autoimmune diseases, or any other condition that could impair the patient’s ability to comply with the study procedures; and prostate biopsy is contraindicated. The clinical and pathological characteristics of all patients are summarized in Table 1.

After histopathological examination, samples were categorized into two groups: 64 (66%) were “PCa” tissue, and 32 (34%) were “non-PCa,” i.e., non-tumorous tissue (benign prostate) obtained from independent and healthy individuals. The average age of the PCa patients was 68 years (ranging from 41 to 82), while the average age of non-PCa patients was 67 years (ranging from 45 to 83).

### 2.2. Risk Factors Identified in Our Patient Cohorts

The median value of the prostate-specific antigen (PSA) in the PCa group was 305 ng/mL, while in the non-PCa group, it was 10.5 ng/mL. The median body mass index (BMI), another risk factor considered for PCa, was 27 (ranging from 17.89 to 36.17) in the PCa group, while in the non-PCa group, it was 25.9 (ranging 22.8 to 31.6). Noticeably, 37 (57.8%) patients of the PCa group were addicted to smoking (*n* = 4; 6.25%), alcohol (*n* = 11; 17.18%), and both alcohol and smoking (*n* = 22; 34.37%). In this group, six patients (9.7%) had a Gleason score < 7; 25 patients (40.3%) had a Gleason score = 7; and 31 patients (50%) had a Gleason score > 7. Additionally, 41 patients (74.5%) were diagnosed with perineural invasion, and 19 patients (38%) were confirmed with distant metastasis (Table 1).

### 2.3. Sequencing Statistics

We performed next-generation sequencing of a panel designed to analyze 15 genes strongly associated with the PCa development from PCa and non-PCa samples. Our 15-gene panel generated 471 amplicons of 200 base pairs on average, with a total length of 57.32 kb and an average coverage of 97.73%. On average, a total of 5,152,539 reads were obtained for each patient, with 95.6% of readings mapped into the target sequences and an average depth of 8214X.

### 2.4. Genomic Alterations

**Figure 1:**
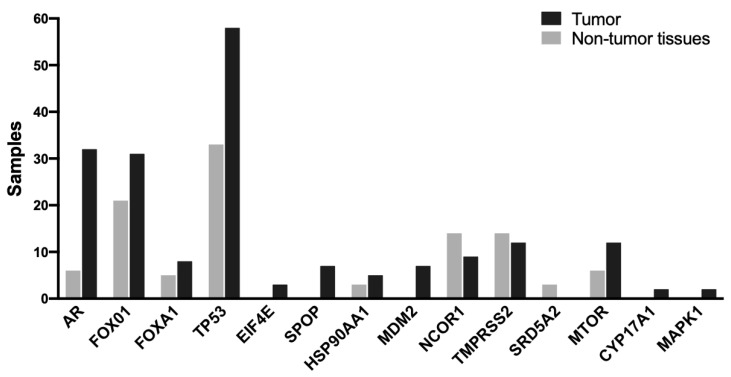
**Frequency of functional mutations in the genes analyzed across biopsies.** *Black bars*, tumors; *Gray bars*, non-PCa tumor biopsies. Missense mutations, nonsense mutations, frameshift deletions, and frameshift insertions were considered. *TP53*, *AR*, *FOXO1*, *MTOR*, and *TMPRSS2* were the genes with mutations in the highest number.

**Figure 2:**
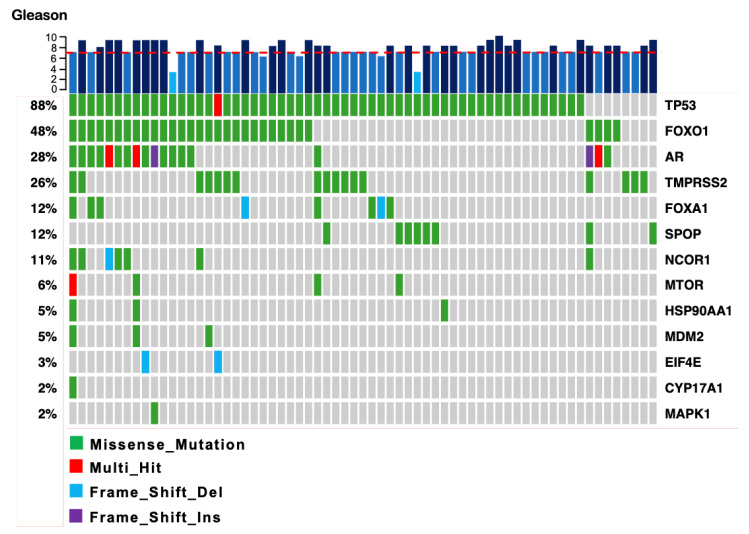
**Number and type of mutations per gene in tumor samples.** *TP53*, *FOXO1*, *AR*, *TMPRSS2*, and *FOXA1* are the genes with the highest number of possible pathogenic mutations. Each square represents a tumor sample. The Gleason score is indicated in *blue bars* on top. Gleason scores above 7 are delimited with a red line. The type of mutations is color-coded on the bottom. Gray squares represent tumor samples without mutations hits.

After variant annotation, a total of 3414 genomic variants were identified, of which 308 were classified as probably pathogenic variants (287 missense, nine frameshift insertion, five non-frameshift deletion, two frameshift deletion, and five nonsense); 909 were synonymous variants; and 2197 were variant of uncertain significance (VUS) (Supplementary Table S2). We plotted the number of functional mutations for each analyzed gene across all samples, both tumor and non-PCa tumor. Interestingly, the genes with the highest number of mutational events were *TP53*, *AR*, and *FOXO1*. Moreover, the genes with the lowest number of mutational events were *EIF4E*, *CYP17A1*, and *MAPK1* (Figure 1). Subsequently, we identified the type of mutations present in every gene in all samples. The top five genes that presented the highest number of mutations were *TP53* (88%), *FOXO1* (48%), *AR* (28%), *TMPRSS2* (26%), and *FOXA1* (12%) (Figure 2). Genes *SRD5A2* and *Nkx3.1* did not present any mutation. The most frequent type of mutations in tumors were missense changes, and the second most frequent were frameshift deletions (Figure 2). No relationship was found between the Gleason score and the number of mutations found in each sample.

**Figure 3:**
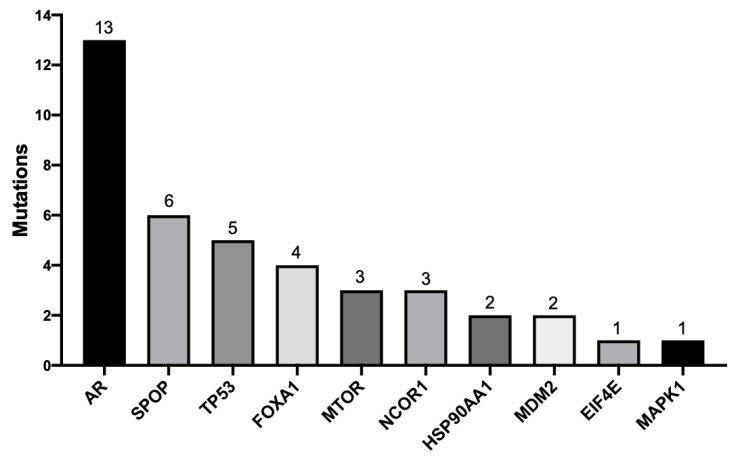
**Number of pathogenic mutations per gene specific of tumor biopsies.** *AR*, *SPOP*, *TP53*, *FOXA1*, and *MTOR* were the most mutated genes. The numbers on top of each bar indicate mutational hits.

We next classified those genetic variants present only in tumor samples according to their biological and clinical significance. Overall, we detected 40 unique pathogenic mutations, 15 unique synonymous mutations (considered benign), and 48 VUS. Within the pathogenic variants, 29 (72.5%) were classified as missense, with one (2.5%) frameshift deletion, five (12.5%) frameshift insertions, three (4.16%) non-frameshift deletions, and two (5%) nonsense (Table 2). Noticeably, *AR* was the gene with the highest number of putative pathogenic mutations with 13 different hits (seven missense mutations, five frameshift mutations, and one nonsense mutation), followed by *SPOP* with six mutations (all missense type), *TP53* with five mutations (four missense and one nonsense), *FOXA1* with four pathogenic variants (two non-frameshift deletion and two missense), *NCOR1* with three mutations (one non-frameshift deletion and two missense), and *MTOR* with three mutations (all missense type) (Figure 3). In *HSP90AA1* and *MDM2* genes, we detected two pathogenic, missense mutations in each gene. In addition, we found a single missense mutation in *MAPK1* and *EIF4E* genes. We did not find genomic variants in *CYP17A1*, *SRD5A2*, *FOXO1*, *NKx3.1*, and *TMPRSSD2* genes that were present only in the PCa samples (Figure 3 and Table 2).

### 2.5. Pathogenic Mutations

After identification of genomic variants, we performed a web-based analysis to predict the potential role of the identified variants by means of SIFT, Polyphen-2, PhyloP, and Grantham algorithms. The most prevalent pathogenic variant found in the PCa patient cohort was *SPOP* c.398T>G (Polyphen-2 score = 1; Grantham score = 155; *n* = 3; 4.68%), followed by *TP53* c.578A>C (Polyphen-2 score = 1; Grantham score = 77; *n* = 2; 3.1%) and *EIF4E* c.144_145delTA (frameshift deletion; Polyphen-2 and Grantham score; *n* = 2; 3.1%). Other pathogenic variants found with a lower frequency were *AR* c.61G>A (SIFT score = 0; Polyphen-2 score = 1; Grantham score = 125; *n* = 1; 1.56%), *FOXA1* c.655C>A (SIFT score = 0; Polyphen-2 score = 1; Grantham score = 110; *n* = 1; 1.56%), *MTOR* c.5705A>T (SIFT score = 0; Polyohen-2 score = 1; Grantham score = 152; *n* = 1; 1.56%), and *NCOR1* p.Gln1602Arg (SIFT score = 0; Polyphen-2 score = 1; Grantham Score = 43; *n* = 1; 1.56%). Among the previously reported genomic variants, seven were confirmed as pathogenic variants, nine as probably pathogenic, and two as probably benign, and two were reported with interpretation conflicts. All pathogenic variants found are summarized in Table 2.

### 2.6. Novel Mutations

Among the 40 genomic mutations identified, we found 19 without a previous report (i.e., novel mutations, specific of our Hispanic cohort), of which 11 were confirmed as pathogenic changes and seven as probably pathogenic, and one variant was classified as probably benign. All novel mutations are summarized in Table 2. The novel mutations were found in *AR*, *FOXA1*, *EIF4E*, *HSP90AA1*, *MAPK1*, *mTOR*, *NCOR1*, and *MDM2*. To gain insight of the novel mutations’ impact on proteins, we performed AlphaFold three-dimensional (3D) structure predictions [46] of those mutations based on the tertiary structures already described.

**Figure 4:**
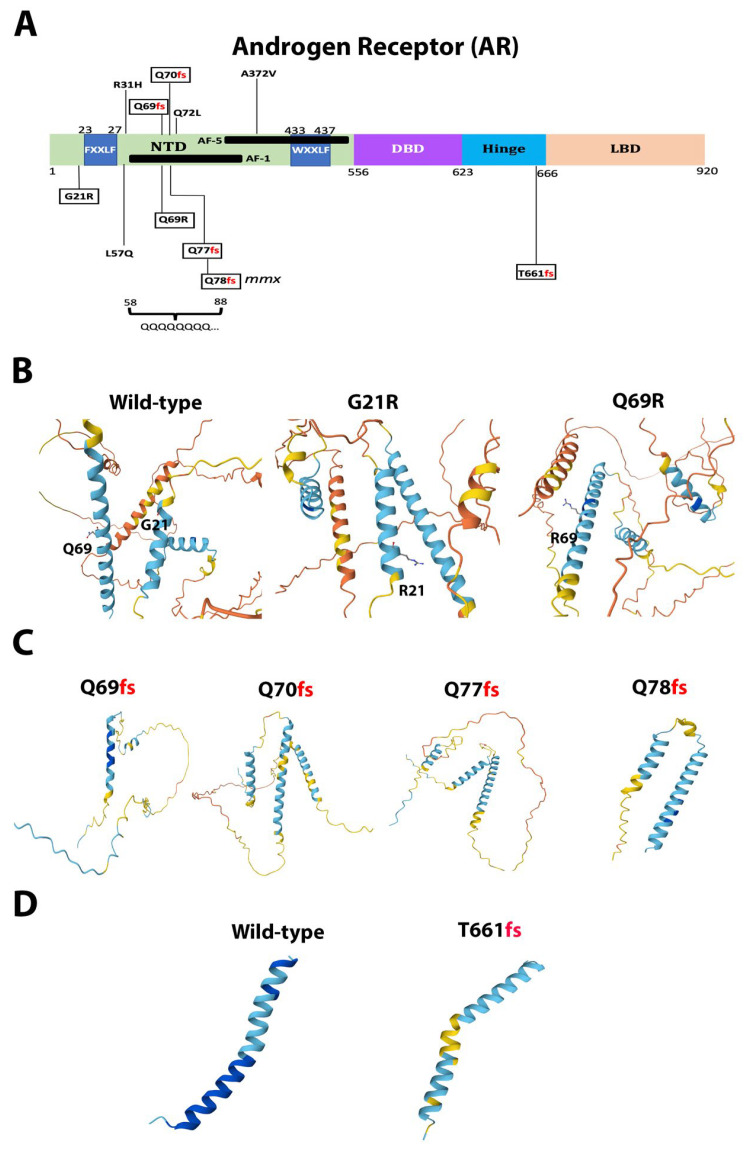
**Functional mutations found in AR in PCa tissues.** (**A**) The AR protein and its domains. The described and novel mutations discovered in this study are shown without and within squares, respectively. *NTD*, amino-terminal domain; *AF-1* and *AF-5*, activation function-1 and -5 motifs, respectively; *FXXLF* and *WXXLF*, LxxLL motifs; *QQQQQQQQ…*, polymorphic region with variable (8–31) number of glutamine residues, spanning amino acids 57–88; *fs*, frame-shift; *DBD*, DNA-binding domain; *LBD*, ligand-binding domain. (**B**,**C**) AlphaFold 3D structure predictions of the NTD, wild-type and the indicated mutant proteins. (**D**) AlphaFold 3D structure predictions of the Hinge domain, wild-type and the indicated mutant protein.

We found seven novel mutations in AR, namely G21R, T66fs, Q69fs, Q69R, Q70fs, Q77fs, and Q78fs (Figure 4A). All of them hit the amino-terminal domain (NTD) of the protein, which is an intrinsically disordered region [34,47]. Within the NTD, *AR* possesses a polymorphic region of polyglutamine (poly Q) encoded by 8–31 repeats of the CAG codon. Changes in the length of the poly Q tract determine the size of the α-helical structure of *AR* [34,47], as well as the transcriptional activity of it [48,49]. T66fs, Q69fs, Q69R, Q70fs, Q77fs, and Q78fs were located within the poly Q tract (Figure 4B). All, except Q69R, were predicted to produce truncated proteins that might act like dominant negative peptides disturbing *AR* transcriptional activity.

**Figure 5:**
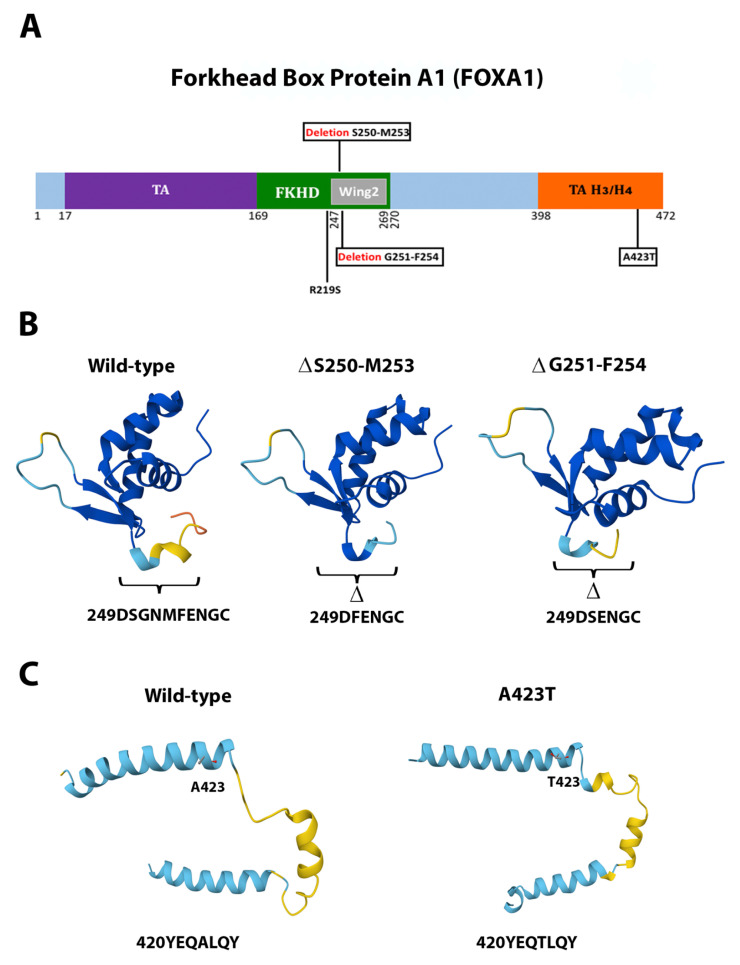
**Functional mutations found in FOXA1 in PCa tissues.** (**A**) The FOXA1 protein and its domains. The described and novel mutations discovered in this study are shown without and within squares, respectively. *TA*, N-terminal transactivation domain; *FKHD*, forkhead DNA-binding domain; *Wing2*, second winged loop; *TA H3/H4*, C-terminal transactivation domain associated with histone H3/H4. (**B**,**C**) AlphaFold 3D structure predictions of the wild-type and mutated proteins. (**B**) Only the FKHD is shown. (**C**) Only TA H3/H4 domain is shown.

Deletions ΔS250-M253 and ΔG251-F254 in FOXA1 lay within the central forkhead DNA-binding domain (Figure 5A). They shortened the coded peptide, from ten to six amino acids-long (249DSGNMFENGC toward DFENGC and DSENGC, respectively), and are predicted to distort the β-sheet of the Wing2 domain [50] (Figure 5B). These deletions are located within the mutational hotspot across cancers that modify the transcriptional activity of FOXA1 [50,51,52,53]. Thus, it is predicted that the deletions we found in this study will also deregulate FOXA1 activity, and that may drive cancer. We also modeled the mutation A423T within the carboxy-terminal transactivation (TA) domain of the protein, which associates with histone H3/H4. This mutation is predicted to cause a spatial distortion forming an extra short α-helix, possibly modifying its protein-protein binding capacities (Figure 5C).

**Figure 6:**
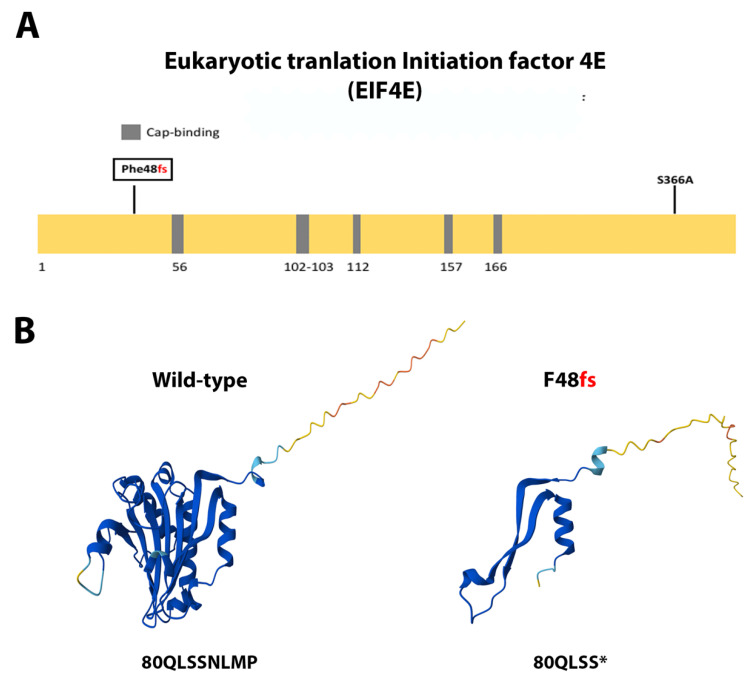
**Functional mutations found in eIF4E in PCa tissues.** (**A**) The eIF4E protein. The described and novel mutations discovered in this study are shown without and within squares, respectively. *Cap-binding*, residues involved in ^7^mGTP (cap)-binding; *fs*, frame-shift. (**B**) AlphaFold 3D structure prediction of both the complete wild-type and mutated proteins. Frameshift mutation F48S is predicted to produce a truncated peptide. An asterisk indicates a premature stop of the protein.

**Figure 7:**
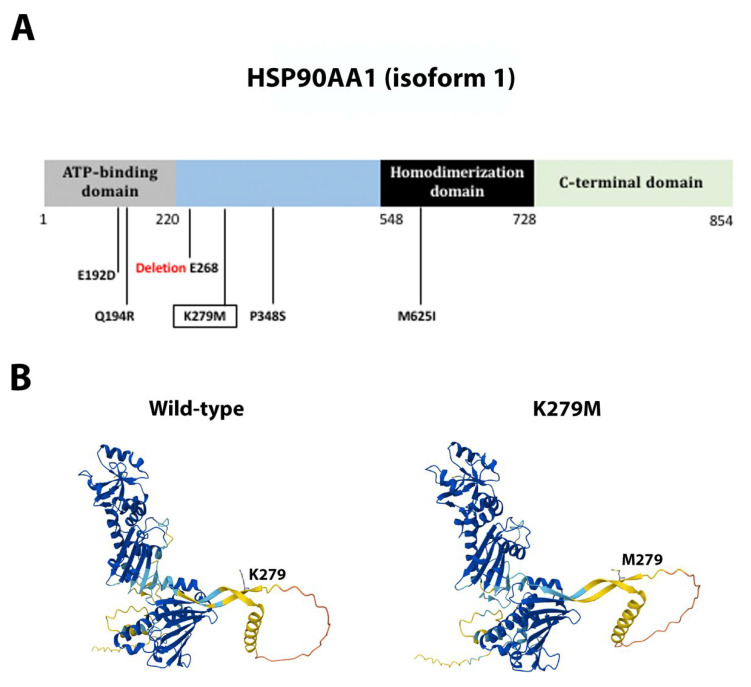
**Functional mutations found in HSP90AA1 in PCa tissues.** (**A**) The HSP90AA1 protein and its domains. The described and novel mutations discovered in this study are shown without and within squares, respectively. (**B**) AlphaFold 3D structure prediction of both the wild-type and mutated proteins.

**Figure 8:**
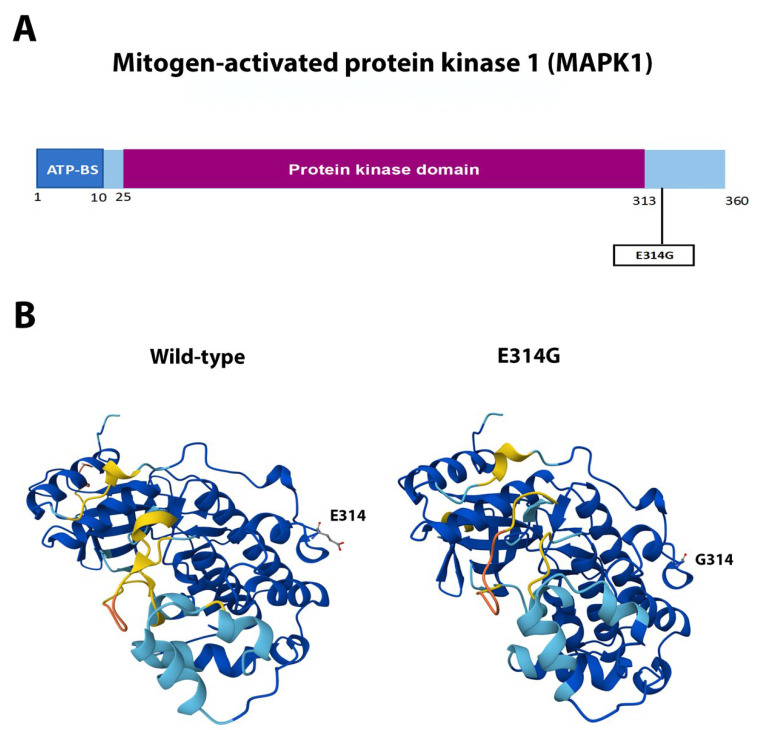
**Functional mutations found in MAPK1 in PCa tissues.** (**A**) The MAPK1 protein and its domains. The described and novel mutations discovered in this study are shown without and within squares, respectively. *ATP-BS*, ATP-binding site. (**B**) AlphaFold 3D structure prediction of both the wild-type and mutated proteins.

eIF4E contains four α-helices intercalated with eight β-sheets forming a cupped-hand-shaped protein [54,55]. Mutation F48 frameshift (Figure 6A) is predicted to produce a truncated protein at the beginning α-helix 3, with a sequence change 80QLSSNLMP toward 80QLSS (Figure 6B). *HSP90AA1* missense, non-conservative mutation K279M (Figure 7A) appears not to significantly alter the overall protein structure [56,57], as it is oriented toward the solvent part of the protein and was located in between the two functional domains, namely the ATP-binding domain and the homodimerization domain (Figure 7B). MAPK1 missense, non-conservative mutation E314G (Figure 8A) is located at the α-helix 14 at the protein carboxi-terminus [58,59], outside the kinase domain and the active site. This mutation is predicted to cause little spatial distortion (Figure 8B).

**Figure 9:**
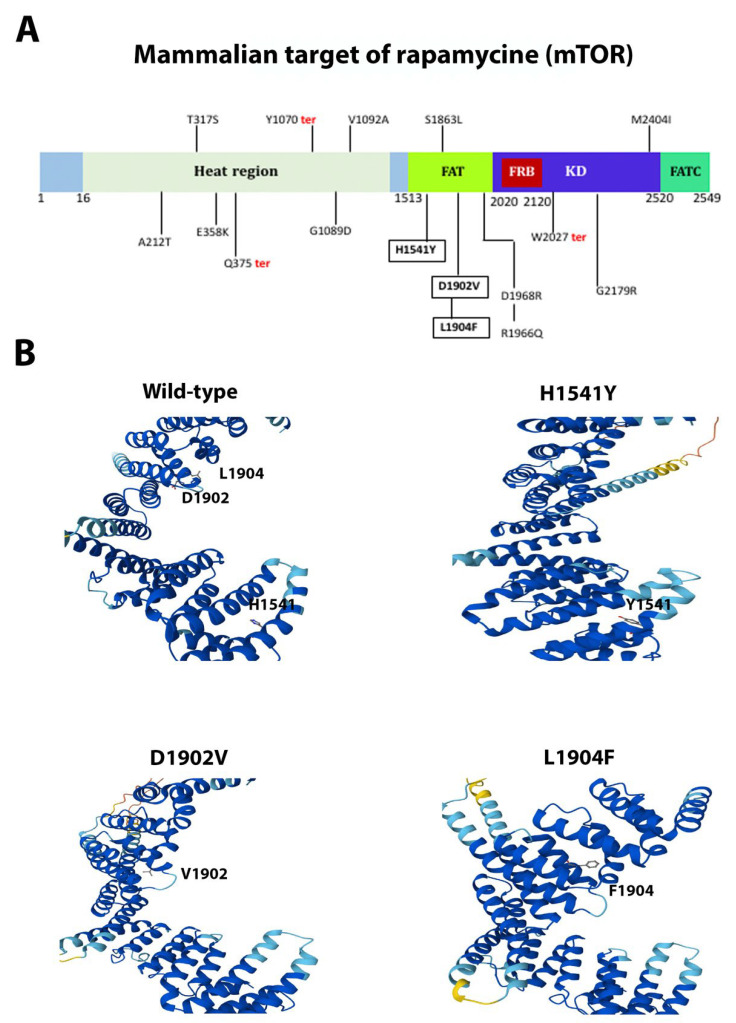
**Functional mutations found in mTOR in PCa tissues**. (**A**) The mTOR kinase and its domains. The described and novel mutations discovered in this study are shown without and within squares, respectively. *FAT*, FRAP, ATM, and TRRAP domain; *FRB*, FKBP12-rapamycin-binding domain; *KD*, kinase domain; *FATC*, FAT domain at the carboxy-terminus. (**B**) AlphaFold 3D structure prediction of the wild-type and mutated proteins. Only the FAT domain is depicted.

**Figure 10:**
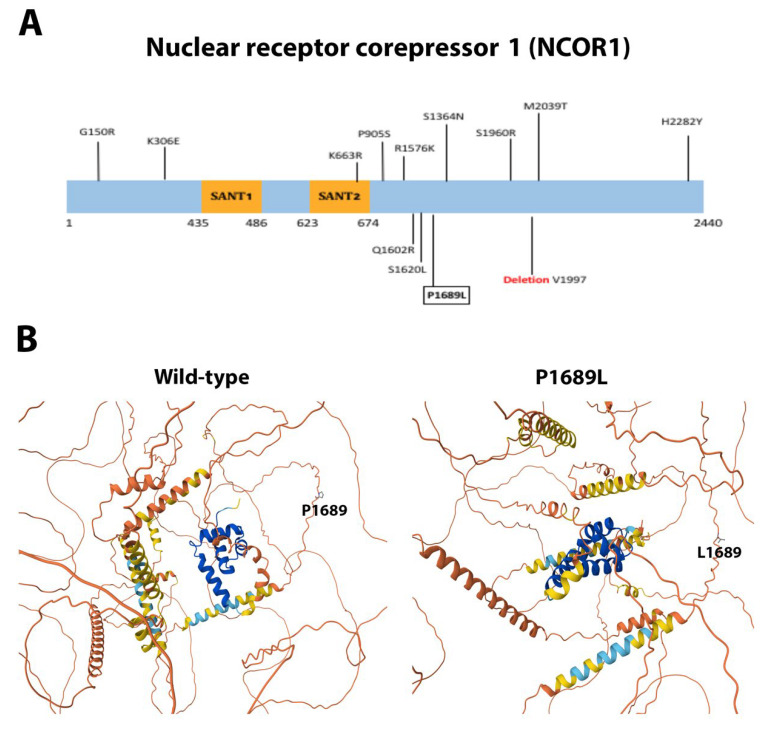
**Functional mutations found in NCOR1 in PCa tissues.** (**A**) The NCOR1 protein and its domains. The described and novel mutations discovered in this study are shown without and within squares, respectively. *SANT1*, component of the deacetylase activator domain; *SANT2*, histone interaction domain. (**B**) AlphaFold 3D structure prediction of both the wild-type and mutated protein.

**Figure 11:**
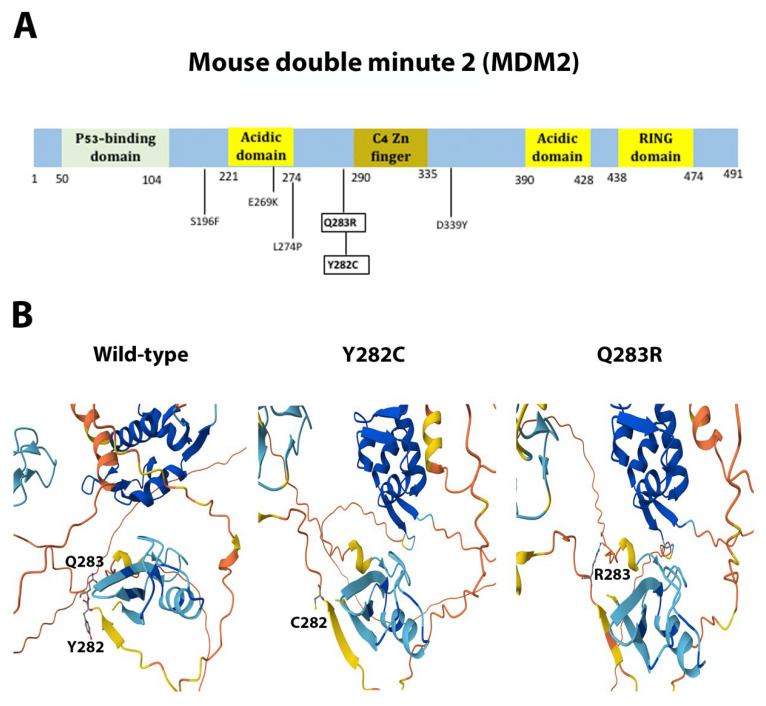
**Functional mutations found in MDM2 in PCa tissues.** (**A**) The MDM2 protein and its domains. The described and novel mutations discovered in this study are shown without and within squares, respectively. (**B**) AlphaFold 3D structure prediction of the wild-type and mutated proteins.

Missense mutations H1541Y (non-conservative), D1902V (non-conservative), and L1904F (conservative) of the kinase mTOR lay within the FAT domain (Figure 9A), a region critical for the catalytic activity of the protein. The FAT domain is a structural “C”-shaped unit composed of α-α-helical repeats forming an α-solenoid wrapping around the kinase domain and clamps onto it [60,61]. The mutations H1541Y and L1904F are predicted to cause slight distortions in α-helix 10 and α-helix 23, respectively, and D1902V makes a disordered structure around its spatial location in α-helix 23 (Figure 9B). In NCOR1, we have described the missense and non-conservative mutation P1689L, located outside the SANT1 and SANT2 motifs (Figure 10A), that promotes histone deacetylation and repression [62]. Since this mutation appeared to be in a predicted flexible and non-structured region, 3D structural predictions indicated little or no spatial change of the protein (Figure 10B). Finally, we described the missense mutations Y282C (conservative) and Q283R (non-conservative) in MDM2. Both changes are located between the acidic domain and the C4 Zn finger domain (Figure 11A). This is an unstructured and flexible region with no reported function [63]. The changes Y282C and Q283R were predicted not to significantly change the shape of this flexible region (Figure 11B).

### 2.7. Association Between Genetic Mutations and Clinicopathologic Characteristics

**Figure 12:**
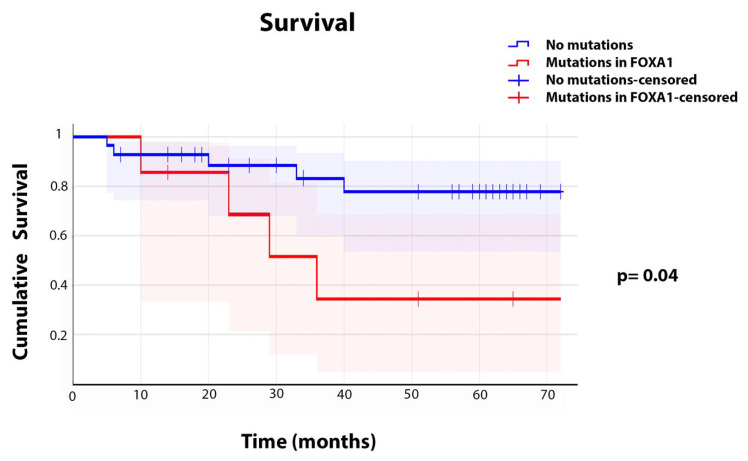
**Kaplan–Meier survival curves comparing patients with and without mutations in *FOXA1***. *Blue line*, patients without mutations; *Red line*, patients carrying mutations. Censored data points are indicated by vertical ticks (blue and red for the respective groups). Patients with mutations in *FOXA1* exhibited significantly decreased overall survival compared to those without mutations (log-rank test, *p* = 0.04).

The prevalence of mutations in our cohort of PCa patients was 40.6% (26 out of 64 patients had one or more mutations). We analyzed the correlation between each mutation found and the overall patient survival included in this study. Interestingly, only the mutations c.655C>A (amino acid change R219S), c.1267G>A (amino acid change A423T), c.749_760delCCGGCAACATGT (deleting amino acids 250–253), and c.752_763delGCAACATGTTCG (deleting amino acids 251–254) found in *FOXA1* showed a significant negative association with the overall patient survival. In order to assess the prognostic impact of *FOXA1* mutations, we performed a Kaplan–Meier survival analysis comparing patients with and without mutations in this gene. As shown in Figure 12, patients harboring *FOXA1* mutations (red line) exhibited a markedly reduced overall survival compared to those without mutations (blue line). The cumulative survival rate declined more rapidly in the mutant group throughout the follow-up period. This difference was statistically significant (log-rank test, *p* = 0.04), indicating that *FOXA1* mutations are associated with an adverse prognosis in this cohort.

We further analyzed the overall survival data of patients with each of the clinical characteristics. We found that those patients who have undergone a prostatectomy had a slightly better prognosis (92% vs. 75%, Supplementary Figure S1A). Similarly, those patients who were treated with radiotherapy had longer survival periods (85% vs. 70%, Supplementary Figure S1B), with statistical significance. In our data, no statistical significance was found between any mutation and the Gleason score (chi-squared test) or PSA level (Wilcox test) for all patients.

## 3. Discussion

### 3.1. Novel Mutations from a Novel Genetic Background

**Figure 13:**
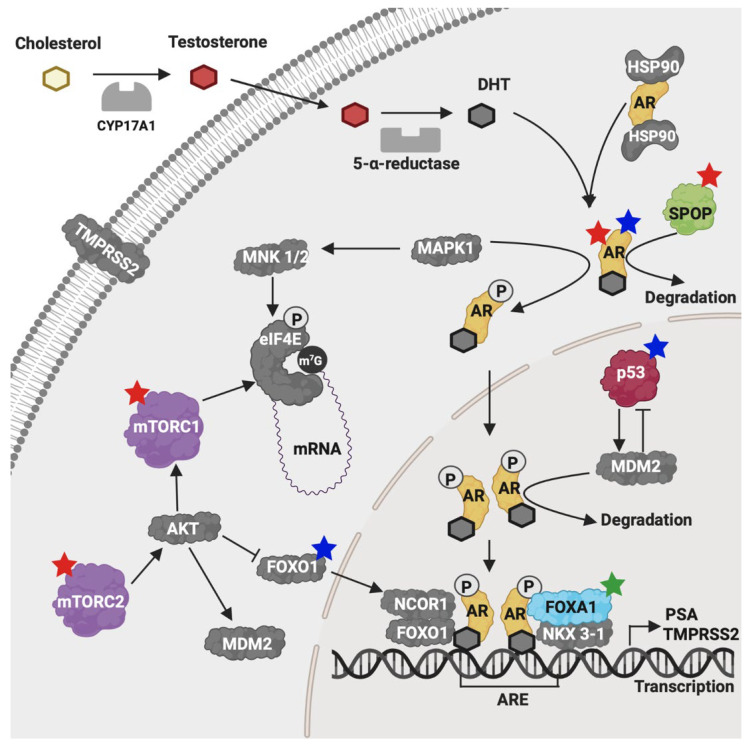
Genes with relevant mutations and their involvement in the AR activation pathway. Proteins encoded by all genes here studied are shown. The top five proteins with the highest rate of pathogenic mutations in tumors are depicted in color. *Blue stars* indicate genes with the highest frequency of mutations across all biopsies; *red stars* indicate genes with the highest number of pathogenic mutations exclusively in tumors; *green stars* indicate mutations with a positive patient survival association. *AR*, androgen receptor; *SPOP*, speckle-type POZ protein; *FOXA1*, Forkhead box protein A1; *MTOR*, mammalian target of rapamycin; *DHT*, dihydrotestosterone; *P*, phosphorylation; *PSA*, prostate specific antigen. Image created with BioRender.com.

PCa displays one of the most significant disparities across Caucasian, Asian, and African descendants, proving that the race-specific genetic background is critical for the PCa development [8,10,11,12,64]. Overall, we identified 3414 genomic mutations utilizing NGS, with a high number of them of uncertain significance (VUS) (64%, 2197 events). Among them, 19 were novel. We analyzed the genomic variants following the American College of Medical Genetics and Genomics (ACMG) recommendations [65], which are based in categorization, annotation, and reporting using clinical, experimental, and bioinformatic evidence [66]. A summary of the most relevant mutations found in this study is depicted in Figure 13.

Our discovery of Hispanic-specific mutations is very relevant, as most PCa research, including genome sequencing, has disproportionally focused on European descendants [4,67,68,69]. Likewise, above 85% of mutations and polymorphisms associated with cancer have been discovered in people with European genetic background. In contrast, only 10% belong to Asians and 5% to African descendants [1,69,70]. Thus, populations with genetic backgrounds from other world regions are either scarce or absent in such studies. This bias toward the European background limits the understanding of the genetic variability of PCa. The Hispanic-specific mutations discovered in this study add key information on the genetic diversity of the mutational landscape in genes critical for PCa. The mutations detected here might have a specific impact on the onset and development of PCa in Hispanics.

The novel mutations discovered in this study are described in Table 2 and are shown within squares in Figure 4A, Figure 5A, Figure 6A, Figure 7A, Figure 8A, Figure 9A, Figure 10A and Figure 11A. Eight of them were in the *AR*, three in *FOXA1*, three in *MTOR*, two in *MDM2*, and only one in *EIF4E*, *HSP90AA1*, *MAPK1*, and *NCOR1*, of which 10 were predicted to be pathogenic changes, nine as putative pathogenic, and one as probably benign. In the following, we will discuss the effect that the novel mutations might have on the protein and PCa.

### 3.2. AR, FOXA1, MTOR, and MDM2 Carried the Highest Number of Novel and Pathogenic Mutations

In our cohort of patients with PCa, *AR*, *FOXA1*, *MTOR*, and *MDM2* showed the highest rate of novel and pathogenic mutations, with 7, 3, 3, and 2, respectively, evidencing their relevance in this type of cancer.

AR malfunctioning triggers primary PCa and metastases and is involved in multiple cellular events, including proliferation, apoptosis, migration, invasion, and differentiation [34,35,40]. The *AR* gene displays a broad spectrum of variability in the mutations landscape across different ethnical backgrounds that might be differently associated with cancer risk [39,41,42,43,44,45,71]. In our cohort, *AR* carried the highest rate of pathogenic mutations in Hispanic patients with PCa (11%). We detected 13 pathogenic mutations (eight novel and four previously reported) in this gene. In our data, patients with *AR* mutations were associated with metastases and a higher Gleason score (≥8, *p* = 0.028). Gaddipati et al. and Thompson et al. also suggested the association of *AR* mutations with advanced stages and metastasis in PCa [72,73]. We also detected the pathogenic change c.170T>A (L57Q) at the NTD, present in advanced PCa [74] male hepatocellular carcinoma [75], medulloblastoma [76], breast cancer [77], and colorectal cancer [78].

All novel *AR* mutations lay within the NTD of the protein, a domain that interacts with many proteins that regulate AR transcriptional activity via the activation domains AF-1 and AF-5 [36]. At the NTD, AR possesses a polymorphic tract of poly Q encoded by 8–31 repeats of the CAG codon. Indeed, changes in the length of the poly Q region determine the transcriptional activity of AR [48,49] and influence the risk of PCa. Short poly Q lengths (≤22 repetitions) are associated with an increased risk of CaP and are more commonly found in African-descendant individuals, less frequent in European individuals, and much less frequent in Asian individuals [42,44,79]. This is consistent with the much higher frequency of PCa observed in African-American men [8,10,11,12,64]. Interestingly, among the novel mutations, six frame shift changes (Q69fs, Q70fs, Q69R, Q77fs, and Q78fs) were located within the poly Q hypervariable region and might produce dominant negative peptides. Due to the functional relevance of both the NTD and the poly Q tract, most probably the mutations we discovered here are relevant for PCa development in Hispanics. Indeed, all novel mutations were classified as probably pathogenic.

### 3.3. Clinical Association of FOXA1 and Patient Survival

*FOXA1* plays a key role in PCa and can also be used to determine distinctive phenotypes in this tumor. High expression of FOXA1 is associated with poor prognosis [80]. Moreover, mutations in *FOXA1* have been frequently associated with poor clinical outcomes in PCa [50,51,52,53,81,82,83,84]. The frequency of mutations in *FOXA1* in PCa is also influenced by the ethnic background [32], with a frequency of 41% in tumors of Asian populations and 4–13% in North Americans, Europeans, and African descendants [21,23,84,85,86,87,88]. In our cohort, we found 12% mutations in this gene.

Interestingly, we discovered that the mutations in *FOXA1* had a significant negative association with the overall patient survival (Figure 12), namely c.655C>A (amino acid change R219S) [52], the novel mutation c.1267G>A (amino acid change A423T), and the novel deletions Δc.749_760delCCGGCAACATGT (deleted amino acids A250-M253) and Δc.752_763delGCAACATGTTCG (amino acid deleted G251-F254), which partially overlapped or clustered with the described deletions ΔM253-N256 [52], ΔF254-E255 [52], the mutations FENG254-7C [89] and G275X [52], and the deletion ΔR265-Q271 [50], within the mutational hotspot second winged loop (Wing2) in the forkhead DNA-binding domain (FKHD; Figure 5B,C). The reported mutations deregulate transcription-causing cellular phenotypes across different cancers [50,51,52,53]. Taken together, these observations support the notion that the *FOXA1* mutations we found play a significant role in the clinical outcome of Hispanic PCa patients.

In PCa progression, the two central signal transduction pathways are Ras/MAPK and PI3K/Akt/mTOR, both converging on eIF4E [90]. mTOR is a central hub in the PI3/Akt/mTOR signaling cascade that controls cell growth and proliferation in response to energy, nutrients, growth factors, and environmental clues. This pathway is often hyperactivated in most tumors, including PCa [61,90,91,92]. Beyond signal transduction, mTOR also plays a role in gene transcription. During PCa, mTOR also directly associates with chromatin in different complexes with transcriptional regulators, including AR, to drive chromatin remodeling and the transcription of genes relevant for cancer progression [93,94,95,96]. Genomic profiling data have shown that mTOR alterations (i.e., mutations and amplifications) occur in 1.78% of PCa cases and 2.93% of in metastatic prostate adenocarcinoma [90]. We have discovered the novel mutations H1541Y within the FAT domain of mTOR. Since the FAT domain associates with different regulator proteins to form the complex mTORC1 and mTORC2, most mutations in the FAT domain deregulate mTOR activity [60,61]. This might also be the case of mutations H1541Y, L1904F, and D1902V.

MDM2 is an oncoprotein inhibitor of p53. MDM2 associates with p53, diminish its transcriptional activity, escorts it from the nucleus to the cytoplasm, and ubiquitylates p53 for degradation by the 26S proteasome [97]. MDM2 also plays p53-independent activities in regulating genomic stability [98,99,100] and stabilizing different mRNAs [101,102]. Malfunctioning of MDM2 is strongly involved in carcinogenesis in many tissues, including prostate, as it also targets AR for degradation [103,104,105,106]. We described the novel mutations Y282C and Q283R within a disordered region [63] with no reported function. The relevance of these mutations in MDM2 function and PCa remains obscure.

### 3.4. Single Novel Mutations in EIF4E, HSP90AA1, MAPK1, and NCOR1

*EIF4E*, *HSP90AA1*, *MAPK1*, and *NCOR1* had one novel mutation in our cohort. eIF4E drives mRNA translation. Moreover, a fraction of the protein is found in the nucleus and cytoplasmic foci, where it mediates mRNA export and storage, respectively [107]. eIF4E is a protooncogene, key for cell signaling in cancer [108], and is overexpressed in different tumors, including PCa [109]. Downregulation of eIF4E reduces the translation of only a defined set of mRNAs involved in cell proliferation and transformation in cells and in mice [110,111,112]. Thus, eIF4E has been proposed as a prime target for anticancer therapies. We described the novel mutation F48fs, which will produce a truncated, non-functional protein. Therefore, this mutation probably reduces the carcinogenic phenotype in prostate tumors.

The chaperone HSP90AA1 plays a pivotal role in helping newly synthesized proteins fold correctly and maintaining protein stability and function [56]. HSP90AA1 interacts with several oncoproteins, including kinases and transcription factors, promoting the development of different tumors, including PCa [113]. In PCa, HSP90AA1 promotes chronic inflammation of fibroblasts through the activation of NF-kB and STAT3, which in turn stimulates PCa progression [114]. N-domain ATP binding and hydrolysis is key for HSP90AA1 activity [57]; thus, most inhibitors of this protein target this domain, which leads to proteasome-mediated degradation of HSP90AA1 partners [56]. We described the novel mutation K279M, laying outside the ATP-binding domain and possibly not leading to significant overall structural changes of the protein. We cannot predict what its effect on cancer might be.

The kinase MAPK1 integrates and transduces multiple signals and is involved in transcription, translation, apoptosis, stress responses, cell proliferation, differentiation, and migration [115]. Aberrant signaling by the MAPK1 cascade plays a crucial role in carcinogenesis [116,117]. In most cancer types (including PCa), the MAPK cascade is hyperactivated, promoting tumor growth, castration-resistant carcinogenesis, and metastasis [118]. Indeed, 25% of the PCa tumors show a presumed actionable lesion in members of the MAPK1 signaling pathways [85]. In our cohort, we found the novel mutation E314G outside the MAPK domain and the active site with non-significant structural and functional changes [58,59] and unknown (if any) relevance for cancer progression.

NCOR1 is a transcriptional repressor that interacts with nuclear hormone receptors, transcription factors (including AR), and chromatin-condensation enzymes [119]. It is also key in oxidative metabolism signaling in the mitochondria [120]. NCOR1 malfunctioning is involved in cell proliferation and carcinogenesis in breast, colorectal, bladder, cervical, and prostate cancers [121,122,123,124,125,126]. NCOR1 genomic alterations have been identified in 5% of PCa cases and have been proposed as a molecular marker of a subtype of PCa [127]. Moreover, NCOR1 downregulation may be predictive of resistance to castration therapy in PCa patients expressing AR [122]. We described the novel mutation P1689L, which hit outside the DNA-binding motifs SANT1 and 2.

## 4. Materials and Methods

### 4.1. Patients and Tissue Samples

The research was performed under the approved protocols by both the Ethics Committee (approval CEI/905/14) and the Research Committee (approval 014/019/IBI) of the National Institute of Cancer, Mexico (Instituto Nacional de Cancerología, Mexico City, Mexico). We confirm that all research was performed in accordance with the guidelines and regulations of this Institute and in accordance with the Declaration of Helsinki. We also confirm that informed consent was obtained from all participants and/or their legal guardians to use their samples for diagnostic and investigation purposes, as well as for publication of the resulting data in an anonymous way.

After obtaining informed written consent, prostate tissue samples were obtained between February 2016 and September 2018 from 96 Mexican patients attending the Department of Oncologic Urology in the National Institute of Cancer (Mexico). The inclusion criteria for patient selection were as follows: patients were born in Mexico with both parents and both grandparents also born in Mexico; blood PSA levels above 4 ng/mL; and presented symptoms related to prostatic pathologies such as dysuria, hematuria, and abnormal digital exam. The exclusion criteria were as follows: patients selected for the study did not receive any previous treatment with androgenic antagonists. Following the sample’s recollection, tissues were examined by the Pathology Department and were classified either as cancerous tissue or non-cancerous tissue. All samples were processed anonymously and stored in liquid nitrogen until nucleic acid extraction.

### 4.2. Genomic DNA Extraction

Nucleic acids for Next Generation Sequencing were obtained from the collected tissues. Genomic DNA (gDNA) was extracted using the Wizard Genomic DNA Purification kit (Promega Corp., Madison, WI, USA) following the manufacturer’s instructions. Extracted gDNA was quantified by real-time PCR reactions using the TaqMan RNase P Detection Reagents kit (ThermoFisher Scientific, Waltham, MA, USA), following the Demonstrated Protocol Sample Quantification for an Ion AmpliSeq Library Preparation (Pub. No. MAN0007732).

### 4.3. Generation of Primer Pools for Target Genes

A panel of 15 genes directly related to the AR function or relevant to prostate cancer were included in this study (Table 3). Genes were selected under two criteria: the importance for the *AR* gene signaling pathway and the number of previous reports where they have been associated with PCa. The sequencing panel was designed using the Ion AmpliSeq Designer tool by ThermoFisher Scientific (Waltham, MA, USA), following the manufacturer’s instructions. Primers were designed using the human genome GRCh38 as a reference (http://www.ncbi.nlm.nih.gov/grc/human accessed on 2 June 2014). The average target size was 57.32 kb, with an average of 471 amplicons and an average of gene coverage of 97.73%.

### 4.4. Amplicon Library Generation

Twenty nanograms of gDNA were used to elaborate amplicon DNA libraries by means of the Ion AmpliSeq Kit for Chef DL8 (ThermoFisher Scientific, Waltham, MA, USA). A total of 12 different libraries (eight samples per library) were elaborated following the Ion AmpliSeq Library Preparation on the Ion OneTouch 2 system. Samples were diluted equimolar in each of the libraries, and 15 uL of each library were charged into the IonChef system. One hundred fifty uL of two primer pools were added per run. Runs were programmed following the manufacturer’s instructions under the following conditions; 2 primer pools; 18 amplification cycles; 4 min of annealing extension time.

### 4.5. Ion Torrent Sequencing

Generated libraries were sequenced using Ion Torrent S5 equipment (Thermo Fisher Scientific, Waltham, MA, USA). Twenty-five μL of each 100 pM library were loaded into the Ion 540 chips and sequenced. Sequencing was performed following the manufacturer’s instructions. A total of 96 samples were sequenced with an average in-deep coverage of 8214X. Sequencing was performed using the human genome GRCh38 as a reference. The Variant Caller plugin included in the Torrent Suite Software was used to identify variations in target regions.

### 4.6. Data Analyses

Ion Torrent Suite V4.0 was used to align raw fastq files to the GRCh38 reference genome and generate VCF files. After processing the results with the Variant Caller plug-in, a database was elaborated with the xls files. The main database categories were locus; type of mutation; genotype; coverage; gene; genomic localization; protein change; codon change; polyphen score; dbsnp; and sample number. Only mutations with a non-synonymous change were selected for subsequent analysis. Selected mutations were separated into two groups depending on the sample’s origin: either “cancerous” or “non-cancerous.” To determine whether there was a significant correlation between mutational burden and any of the tumor characteristics, we calculated the Pearson correlation coefficient among the number of somatic mutations in each sample, with each of the samples’ clinical characteristics. All statistical analyses were performed in R 3.4.2 (R Core Team, Vienna, Austria).

### 4.7. Variant Classification

Sequence variants detected in this study were identified according to the human genome GRChg38 as a reference using the mutation databases (http://androgendb.mcgill.ca/ and http://www.hgmd.cf.ac.uk/ac/all.php accessed on 2 June 2025), SNP database (https://www.ncbi.nlm.nih.gov/snp/ accessed on 2 June 2025), and Genome Aggregation Database (GnomAD, https://gnomad.broad.institute.org accessed on 2 June 2025). Variants were classified according to the classification guidelines of the International Agency for Research on Cancer (IARC). The clinical interpretation of each variant and their association with a disease is described in ClinVar (https://www.ncbi.nlm.nih.gov/clinvar/ accessed on 2 June 2025), Varsome (https://varsome.com/ accessed on 2 June 2025), and or COSMIC (https://cancer.sanger.ac.uk/cosmic accessed on 2 June 2025) [128]. Missense, nonsense, frame-shift deletion, frame-shift insertion mutation, non-frameshift deletion, splicing variants, and mutations affecting protein function were classified as pathogenic variants. Additionally, we performed a web-based analysis to predict the potential role of the identified variants employing SIFT, Polyphen-2, PhyloP, and Grantham algorithms.

### 4.8. Three-Dimensional (3D) Structure Prediction

Protein 3D structures were predicted by Alphafold2 [46] using the Worldwide Protein Data Bank (PDB) reports. Images were visualized using PyMOL (Schrödinger, L., & DeLano, W. (2020). *PyMOL*. Retrieved from http://www.pymol.org/pymol accessed on 1 May 2025).

## 5. Conclusions

We described 3414 novel somatic mutations in PCa samples, highlighting both the mutational heterogeneity of prostate tumors and the ethnical component of a scarcely studied genetic background. Nineteen of them were novel changes. Of particular interest were those mutations found in FOXA1, which might be used as novel markers for PCa prognosis, at least for Hispanic men.

## Figures and Tables

**Table 1:** Clinical description of the patients analyzed in this study.

Characteristic	Tumor Samples *n* = 64 (66.6%)	Non-Tumor Samples *n* = 32 (33.4%)
**Age**	**≤50:** 4 (6.25%)	≤50: 2 (6.25%)
	>50: 60 (93.75%)	>50: 30 (93.75%)
	*Median* = 68-years-old	*Median* = 6-years-old
**PSA at diagnosis**	≤10 ng/mL: 11 (17.18)	≤10 ng/mL: 18 (56.25%)
	10.1–20 ng/mL: 11 (17.18%)	10.1–20 ng/mL: 11 (34.37%)
	>20 ng/mL: 42 (65.6%)	>20 ng/mL: 3 (9.37%)
	Median = 305 ng/mL	*Median* = 10.5 ng/mL
**Body mass index (BMI)**	<18.5: 2 (3.12%)	<18.5: 5 (15.62%)
	18.5–24.9: 10 (15.62%)	18.5–24.9: 10 (31.25%)
	25–29.9: 26 (40.65%)	25–29.9: 6 (18.75%)
	≥30: 11 (17.18%)	≥30: 6 (18.75%)
	*Without data:* 15 (23.43%)	*Without data:* 5 (15.62%)
	*Median* = 27	Median = 25.9
**Addictions**	*Smoking:* 4 (6.25%) *Alcohol:* 11 (17.18%) *Smoking and alcohol:* 22 (34.37%) *Without data:* 27 (42.18%)	Without data
**Gleason score**	**<7:** 6 (9.37%) **=7:** 25 (39.06%) **>7:** 31 (50%) Without data: 2 (3.12%)	NA
**Perineural invasion**	*Yes:* 41 (64.06%) *No:* 15 (23.43%) *Without data:* 2 (3.12%)	NA
**Metastasis**	*Yes:* 19 (29.68%) *No:* 31 (48.43%) *Without data:* 14 (21.87%)	NA
**Nationality**	100% Mexicans with all four Mexican grandparents	100% Mexicans with all four Mexican grandparents

**Table 2:** Pathogenic and probably pathogenic mutations identified in the cohort of patients diagnosed with prostate cancer from this study.

Gen	Mutation Type	Change in DNA	Change in Protein	Status ^a^	Functional Prediction ^b^
*AR*	Frameshift Insertion	c.230_231insA	p.Gln78fs	Novel	Probably pathogenic
*AR*	Frameshift Insertion	c.203_204insA	p.Gln69fs	Novel	Probably pathogenic
*AR*	Frameshift Insertion	c.206_207insA	p.Gln70fs	Novel	Probably pathogenic
*AR*	Missense	c.61G>A	p.Gly21Arg	Novel	Pathogenic
*AR*	Frameshift Insertion	c.227_228insA	p.Gln77fs	Novel	Probably pathogenic
*AR*	Missense	c.206A>G	p.Gln69Arg	Novel	Probably pathogenic
*AR*	Frameshift Insertion	c.1981_1982insAAAA	p.Thr661fs	Novel	Probably pathogenic
*AR*	Missense	c.1115C>T	p.Ala372Val	Previously reported	Probably pathogenic
*AR*	Missense	c.170T>A	p.Leu57Gln	Previously reported	Probably benign
*AR*	Missense	c.92G>A	p.Arg31His	Previously reported	Pathogenic
*AR*	Missense	c.215A>T	p.Gln72Leu	Previously reported	Probably benign
*EIF4E*	Frameshift Deletion	c.144_145delTA	p.Phe48fs	Novel	Probably pathogenic
*FOXA1*	Missense	c.655C>A	p.Arg219Ser	Previously reported	Pathogenic
*FOXA1*	Missense	c.1267G>A	p.Ala423Thr	Novel	Pathogenic
*FOXA1*	Non-frameshift Deletion	c.752_763delGCAACATGTTCG	p.Gly251_Phe254del	Novel	Probably pathogenic
*FOXA1*	Non-frameshift Deletion	c.749_760delCCGGCAACATGT	p.Ser250_Met253del	Novel	Probably pathogenic
*HSP90AA1*	Missense	c.836A>T	p.Lys279Met	Novel	Pathogenic
*HSP90AA1*	Missense	c.581A>G	p.Gln194Arg	Previously reported	Pathogenic
*MAPK1*	Missense	c.941A>G	p.Glu314Gly	Novel	Pathogenic
*MDM2*	Missense	c.845A>G	p.Tyr282Cys	Novel	Pathogenic
*MDM2*	Missense	c.848A>G	p.Gln283Arg	Novel	Probably benign
*MTOR*	Missense	c.5710C>T	p.Leu1904Phe	Novel	Pathogenic
*MTOR*	Missense	c.4621C>T	p.His1541Tyr	Novel	Pathogenic
*MTOR*	Missense	c.5705A>T	p.Asp1902Val	Novel	Pathogenic
*NCOR1*	Missense	c.5066C>T	p.Pro1689Leu	Novel	Pathogenic
*NCOR1*	Non-frameshift Deletion	c.5989_5991delGTT	p.Val1997del	Previously reported	Probably pathogenic
*NCOR1*	Missense	c.4805A>G	p.Gln1602Arg	Previously reported	Probably pathogenic
*SPOP*	Missense	c.397T>G	p.Phe133Val	Previously reported	Probably pathogenic
*SPOP*	Missense	c.469C>T	p.Leu157Phe	Previously reported	VUS
*SPOP*	Missense	c.398T>C	p.Phe133Ser	Previously reported	Probably pathogenic
*SPOP*	Missense	c.398T>G	p.Phe133Cys	Previously reported	Probably pathogenic
*SPOP*	Missense	c.305T>G	p.Phe102Cys	Previously reported	Probably pathogenic
*SPOP*	Missense	c.304T>G	p.Phe102Val	Previously reported	Probably pathogenic
*TP53*	Missense	c.524G>A	p.Arg175His	Previously reported	Pathogenic
*TP53*	Missense	c.578A>C	p.His193Pro	Previously reported	Pathogenic
*TP53*	Missense	c.1096T>G	p.Ser366Ala	Previously reported	VUS
*TP53*	Nonsense	c.916C>T	p.Arg306Ter	Previously reported	Pathogenic
*TP53*	Missense	c.808T>A	p.Phe270Ile	Previously reported	Pathogenic

^a^ Novel mutations, exclusive from Mexican population. ^b^ Defined by ClinVar (https://www.ncbi.nlm.nih.gov/clinvar/ (accessed on accessed on 2 June 2025)), Varsome (https://varsome.com/ (accessed on 2 June 2025)), and or COSMIC (https://cancer.sanger.ac.uk/cosmic (accessed on 2 June 2025)) and references therein.

**Table 3:** Fifteen-gene panel associated with the *AR* pathway in prostate cancer.

Gene Name	Chromosome	Chr. Start	Chr. End	Num. Amplicons	Total Bases	Covered Bases	Missed Bases	Coverage (%)
*MTOR*	1	11107479	11259414	70	8220	8183	37	99.5
*CYP17A1*	10	102830696	102837366	14	1607	1607	0	100
*MDM2*	12	68808472	68839854	17	1604	1604	0	100
*FOXO1*	13	40559517	40666217	10	1988	1500	488	75.5
*HSP90AA1*	14	102081706	102139409	20	2685	2685	0	100
*FOXA1*	14	37591359	37594977	9	1439	1296	143	90.1
*TP53*	17	7669603	7676599	14	1383	1307	76	94.5
*SPOP*	17	49600372	49622815	9	1215	1215	0	100
*NCOR1*	17	16032290	16194574	73	7829	7829	0	100
*SRD5A2*	2	31526190	31580905	8	815	804	11	98.7
*TMPRSS2*	21	41466136	41508009	16	1730	1693	37	97.9
*MAPK1*	22	21769198	21867445	11	1163	1160	3	99.7
*EIF4E*	4	98881022	98929117	10	915	915	0	100
*NKX3-1*	8	23681215	23682894	5	725	700	25	96.6
*AR*	X	67545141	67723846	19	2873	2873	0	100

## Data Availability

The mutations data generated in this study have been submitted to the Gene Bank Submission number SUB15257899 (submission in progress). They are available also as Supplemental Table S1.

## Supplementary Materials

The following supporting information can be downloaded at: https://www.mdpi.com/article/10.3390/ijms26188758/s1.
