# Supplementary figures and images for: Deep Sequencing Reveals Novel Mutations in Androgen Receptor-Related Genes in Prostate Cancer
Source: Int J Mol Sci. 2025 Sep 9;26(18):8758. doi: 10.3390/ijms26188758 (PMC12469278; doi:10.3390/ijms26188758)

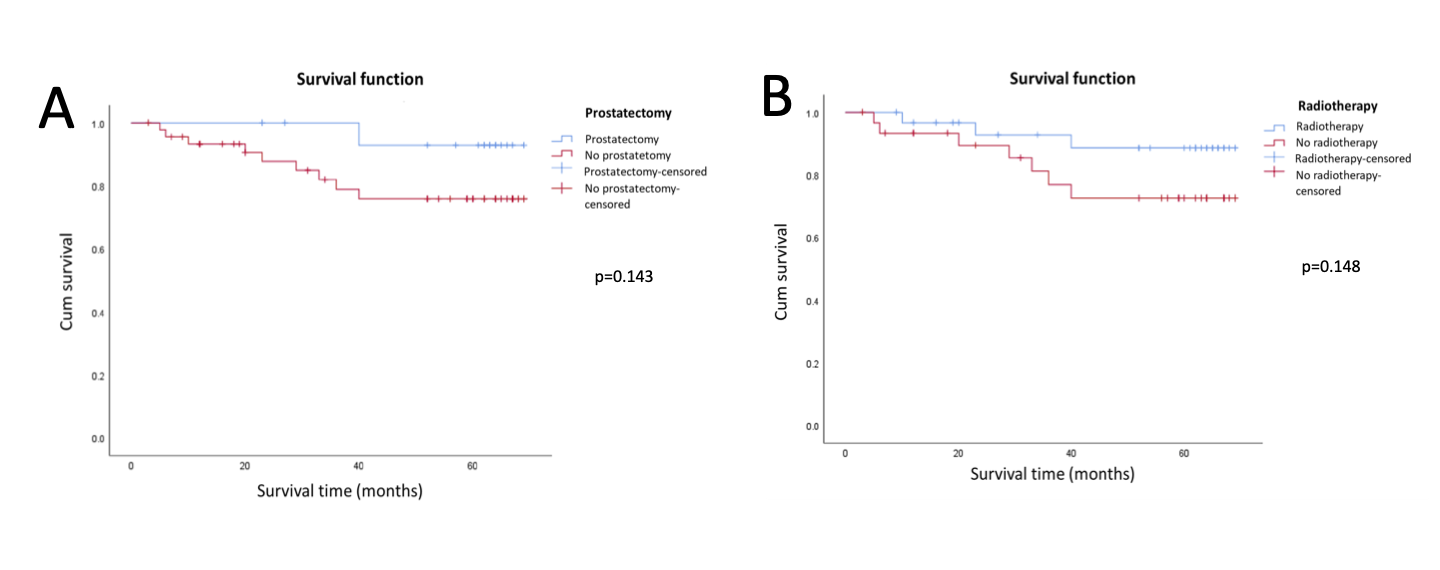

Supplement: Supplementary file 1 [file ijms-26-08758-s001.zip › Suppl 1.tiff]
